# Implementation of the ABL-90 blood gas analyzer in a ground-based mobile emergency care unit

**DOI:** 10.1186/s13049-015-0134-y

**Published:** 2015-07-30

**Authors:** Søren Mikkelsen, Jonathan Wolsing-Hansen, Mads Nybo, Christian Ulrik Maegaard, Søren Jepsen

**Affiliations:** Department of Anaesthesiology and Intensive Care Medicine, Mobile Emergency Care Unit, Odense University Hospital, Odense, Denmark; Institute of Technology and Innovation, University of Southern Denmark, Odense, Denmark; Department of Clinical Biochemistry and Pharmacology, Odense University Hospital, Odense, Denmark; Facilities Management, Odense University Hospital, Odense, Denmark

**Keywords:** Prehospital blood gas analysis, Diagnostic aids on the prehospital scene

## Abstract

Point-of Care analysis is increasingly being applied in the prehospital scene. Arterial blood gas analysis is one of many new initiatives adding to the diagnostic tools of the prehospital physician. In this paper we present a study on the feasibility of the Radiometer ABL-90 in a ground-based Mobile Emergency Care Unit and report on some clinical situations in which the apparatus has proven beneficial.

Point-of-Care analysis and treatment is increasingly being applied in the prehospital setting. Obtaining an electrocardiogram and initiating thrombolysis is by now an established part of the prehospital treatment in coronary occlusion [1]. Increasingly, ultrasound is applied prehospitally [2, 3] and prehospital computerized tomography of the head and ensuing thrombolysis in case of stroke is probably approaching as a treatment modality [4]. Prehospital analysis of blood samples for selected parameters is already applied in some circumstances, most notably measurement of blood glucose, but also troponin and lactate are measured prehospitally [5, 6].

**Fig. 1:**
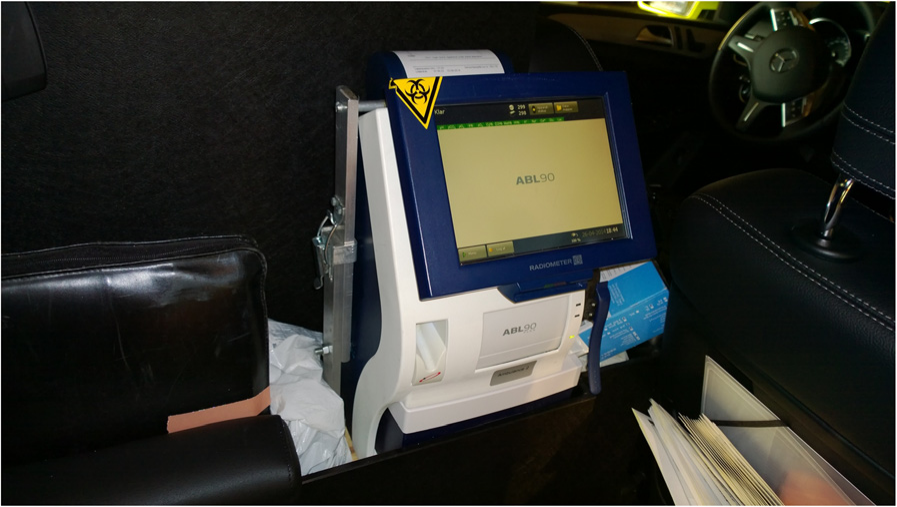
The ABL-90 is secured in its cradle situated to the left of the observers seat behind the physician´s seat

However, full blood gas analysis in prehospital services requires a more extensive set-up than previously described. Within the hospital, the ABL-90 (Radiometer, Denmark) blood gas analyzer has proven fast and reliable. The ABL-90 is portable and data can be sent to the patient data management systems. An internal battery provides back-up when disconnected from the inverter. The apparatus thus would appear suitable for prehospital use.

We report a study on the feasibility of the ABL-90 implemented in the anaesthesiologist staffed Mobile Emergency Care Unit (MECU) in Odense, Denmark.

Initially, the vehicle was equipped with an inverter capable of supplying the ABL-90 with 90 Watts and 230 Volts alternating current. Following that, the susceptibility of the ABL-90 towards vibrations and gravitational forces was assessed. A dummy with dimensions and weight identical to the ABL-90 was constructed and mounted in the vehicle. An accelerometer was placed on the dummy, measuring g-forces during several emergency MECU runs. Furthermore, the shock response spectrum of the unit was calculated. The extent of g-forces exerted and the shock response spectrum measured confirmed the possible functionality of the ABL-90 following the physical impact endured during the emergency runs, and further testing was carried out.

Although g-forces exerted were rather low (1.5–1.95 g in all directions), initial findings demonstrated that the ABL-90 was unreliable. An inlet of the apparatus proved to be responsible for failure, disconnecting the inlet from the calibration system. A re-designed cradle with added cushioning and a newly constructed sample inlet, designed to minimize the risk of a discontinued connection between the sample inlet and solution pack corrected this issue enabling the ABL-90 to function flawlessly. In order to ensure correct results, validation of the blood sample analyses was performed by the Department of Clinical Biochemistry at Odense University Hospital. A maintenance program was also established ensuring that a supplementary ABL-90 is always accessible should maintenance or replacement of solution packs require the unit to be disassembled.

For a period of 18 months the ABL-90 has been functioning in the MECU in Odense, Denmark Fig 1.

The ability to perform rapid, accurate arterial blood gas analysis at the scene has significantly improved the diagnostic possibilities of the MECU, as the diagnosis of several conditions in which a fast diagnosis and subsequent therapy is vital, thus has been made possible.

Among many clinical scenarios where arterial blood gas analyses are performed at the scene, some of the special conditions where the results have proven relevant have been:

In patients with presumed septic shock or septicaemia, analysis of lactate and pH in many cases has enabled the MECU to establish the diagnosis prehospitally, to obtain blood cultures and to initiate antibiotic therapy at the scene.

In several cases, patients suspected of exposure to Carbon monoxide poisoning have been released at the scene thus obviating admission to hospital once blood gas analysis has rejected the presence of Carboxyhemoglobin.

In other related cases, patients exposed to indoor fires with potential for cyanide formation have been diagnosed as the shift to anaerobic metabolism caused by cyanide has been detected by lactic acidosis.

In patients with exacerbation of chronic obstructive pulmonary disease, rapid blood gas analysis has helped the physician at the scene to better direct patient therapy.

Also, the blood gas analyser is helpful in adjusting the level of respiratory support in intubated patients with elevated intracranial pressure, in which the CO_2_-level should be targeted within rather narrower limits than interpretation of end-tidal CO_2_ measurement allows.

As the apparatus has the potential of transmitting prehospital results to intra-hospital patient data management systems it facilitates improved diagnostic and treatment possibilities, both prehospitally and in preparing emergency departments for patients before their arrival at the departments. Transmission of data to the receiving emergency department enables these departments to increase their levels of preparedness and thereby initiate the correct treatment faster.

The direct costs associated with the technology are acquisition of the apparatus. The retail price is EUR 15.000 per unit, while indirect costs (including reagents, syringes etc.) amount to EUR 11.500 per year. Implementation is relatively straightforward. Only minute changes have been made in the MECU to make the ABL-90 suitable for the unit.

## Conclusion

We have successfully introduced the possibility of acquiring full blood gas analyses prehospitally in a mobile emergency care unit.
